# The Anthelmintic Activity of Stonefish (*Synanceia* spp.) Ichthyocrinotoxins and Their Potential as Novel Therapeutics

**DOI:** 10.3390/toxins17020066

**Published:** 2025-02-02

**Authors:** Danica Lennox-Bulow, Jamie Seymour, Alex Loukas, Michael Smout

**Affiliations:** Australian Institute of Tropical Health and Medicine (AITHM), James Cook University, Cairns, QLD 4870, Australia

**Keywords:** novel compounds, stonefish, toxins, anthelmintic, therapeutics, ichthyocrinotoxin

## Abstract

Parasitic gastrointestinal worms (i.e., helminths) remain a significant global health and economic burden. The increasing inefficacy of current anthelmintic drugs against parasitic diseases necessitates the discovery of novel therapeutic options. This study investigated the anthelmintic properties and therapeutic potential of stonefish ichthyocrinotoxins (i.e., secreted skin toxins). xWORM (xCELLigence Worm Real-Time Motility Assay) was used to evaluate the anthelmintic activity of ichthyocrinotoxins from two stonefish species, *Synanceia horrida* (Estuarine Stonefish) and *Synanceia verrucosa* (Reef Stonefish), against the infective third-stage larvae of *Nippostrongylus brasiliensis* (Rodent Hookworm). Both toxins demonstrated potent anthelmintic effects, with *S. horrida* ichthyocrinotoxin exhibiting greater potency (IC_50_ = 196.0 µg/mL) compared to ichthyocrinotoxin from *S. verrucosa* (IC_50_ = 329.7 µg/mL). Fractionation revealed that the anthelmintic activity of *S. verrucosa* is likely driven by synergistic interactions between the large (>3 kDa) and small (<3 kDa) components. In contrast, the small components isolated from *S. horrida* ichthyocrinotoxin were responsible for the majority of the observed activity, making them a more attractive therapeutic candidate. Furthermore, despite the cytotoxicity of crude *S. horrida* ichthyocrinotoxin against human skin and bile duct cell lines, the isolated small components exhibited potent anthelmintic effects (IC_50_ = 70.5 µg/mL) with negligible cytotoxicity (<10% decrease in survival at 100 µg/mL). While further research is necessary to fully characterise these compounds and assess their clinical suitability, this study highlights the potential of stonefish ichthyocrinotoxins as a novel source of anthelmintic therapeutics.

## 1. Introduction

Parasitic worms (or helminths) infect almost all taxa across the animal kingdom [1]. It is estimated that over two billion people, or a little over 24% of the global population, are currently infected with helminths [2,3]. The highest prevalence of helminth infections in humans is found in developing countries, particularly in places with poor sanitation and limited access to healthcare [2,3]. Different helminths cause distinct pathophysiological conditions, depending on the niches they occupy, including intestinal issues (i.e., diarrhoea, abdominal pain), organ damage (i.e., blindness, kidney disease), dermatological conditions (i.e., skin penetration, skin rashes), malnutrition, and impaired growth and physical development [4,5]. Beyond their toll on health and wellbeing, helminths also impose substantial economic losses, particularly in agricultural and aquacultural sectors, by compromising the productivity of livestock and the market value of animal products [6,7,8]. Such production losses can also affect consumers, as reduced supply can strain the market, which in turn, drives up retail prices [8,9].

The devastating impact of helminths on health and the economy is further compounded by the emergence of singular or multi-drug resistance across numerous parasite species. It is now well-established that resistance has developed against all classes of anthelmintic drugs used against helminth species that infect livestock [10]. Moreover, within the last decade, cases of drug resistance have also emerged for helminth species that infect companion animals [11,12], and concerningly, humans [13,14]. Novel anthelmintic treatment options with distinct mechanisms of action are thus urgently needed to address this issue [15,16,17]. Nature has already provided a profusion of modern therapeutics, including analgesics (i.e., penicillin, opium, and aspirin), decongestants (i.e., pseudoephedrine/Sudafed), cholesterol-lowering medications (i.e., statins), anthelmintics (i.e., avermectin), and much more [15,18]. Yet, a vast reservoir of unexplored compounds remains, some of which are likely to have therapeutic potential. Animal toxins, which include venoms and poisons, are particularly promising for biodiscovery due to their highly diverse, dynamic, and often complex composition [19].

Stonefish (*Synanceia* spp.) stand out as being one of few organisms known to possess two distinct types of toxins: a potent defensive venom associated with each of their dorsal spines, and a secreted skin toxin called ichthyocrinotoxin [20,21]. While stonefish are widely distributed throughout the shallow, coastal waters of the Indo-Pacific, two species inhabit Queensland waters: *Synanceia horrida* (Estuarine Stonefish), which are primarily found inshore amongst rocky intertidal areas and estuaries, and *Synanceia verrucosa* (Reef Stonefish), which are commonly associated with coral reefs [22]. Stonefish generally adopt a cryptic, benthic, and predominantly sedentary lifestyle, a strategy that likely assists with prey capture and predator avoidance [22,23]. However, the intimate association that stonefish have with the benthos, combined with their lack of squamation (i.e., scales) [20,22], should render these animals highly susceptible to parasites, particularly those, such as helminths, that burrow through the skin.

Helminths are ubiquitous in the aquatic realm, and as such, their occurrence in fish is common and widely reported, particularly for species targeted or reared for consumption [24,25]. Yet, members of the genus *Synanceia* (including stonefish) appear to harbour a surprisingly low diversity of internal parasite taxa [26]. One hypothesis for the paucity of internal parasites in stonefish may be that their ichthyocrinotoxins afford the skin some protection [27]. Although the role of stonefish ichthyocrinotoxins in parasite defence is currently unknown [28], a previous study on ichthyocrinotoxic *Gobiodon* species supports the hypothesis that some ichthyocrinotoxins may aid in preventing parasitism [29]. This study observed that *Gobiodon* ichthyocrinotoxins influenced the attachment sites of external parasites (gnathiid isopods), with parasites demonstrating a preference for areas, such as the fins, where the toxin glands were less abundant [29].

Furthermore, a previous study demonstrated the broad-spectrum toxicity of stonefish ichthyocrinotoxins against invertebrates. Specifically, *S. horrida* ichthyocrinotoxin was found to inhibit the cilia of invertebrates, including those associated with mussel gills, and protozoan locomotory appendages [20]. The toxin was also found to increase the tone of barnacle depressor scutorum rostralis muscles (i.e., the muscle that controls the scuta lips) for up to one hour after immersion [20]. Additionally, while ichthyocrinotoxins secreted by other fish commonly exhibit both ichthyotoxic and hemotoxic properties [28], stonefish ichthyocrinotoxins do not appear to follow suit. Ichthyocrinotoxins from *S. horrida* were found to demonstrate negligible protease, haemolytic and haemagglutinating activity, and were only mildly toxic to mosquito fishes and blennies [20]. However, *S. horrida* ichthyocrinotoxin was found to inhibit contractions in guinea pig ileum and vas deferens [20,30]. This suggests that while the toxin may have some effect on vertebrates, it appears to be more potent against invertebrates, which potentially include helminths. Moreover, the apparent limited toxicity of stonefish ichthyocrinotoxins to vertebrates warrants further investigation into its therapeutic potential.

Therefore, the aim of this study was to investigate the anthelmintic properties of stonefish (*Synanceia* spp.) ichthyocrinotoxins as a possible explanation for the paucity of endoparasites observed in these animals. Furthermore, this study evaluated the therapeutic potential of stonefish ichthyocrinotoxins by (1) identifying the components responsible for the toxin’s anthelmintic activity against *Nippostrongylus brasiliensis* L3 (infective Rodent Hookworm larvae), and (2) determining the cytotoxicity of these components against various mammalian cell lines (human skin cells, human bile duct cells, as well as human and rat red blood cells) in vitro.

## 2. Results

### 2.1. Assessing the Toxicity of Crude Stonefish Ichthyocrinotoxins to Infective Rodent Hookworm Larvae

**Figure 1:**
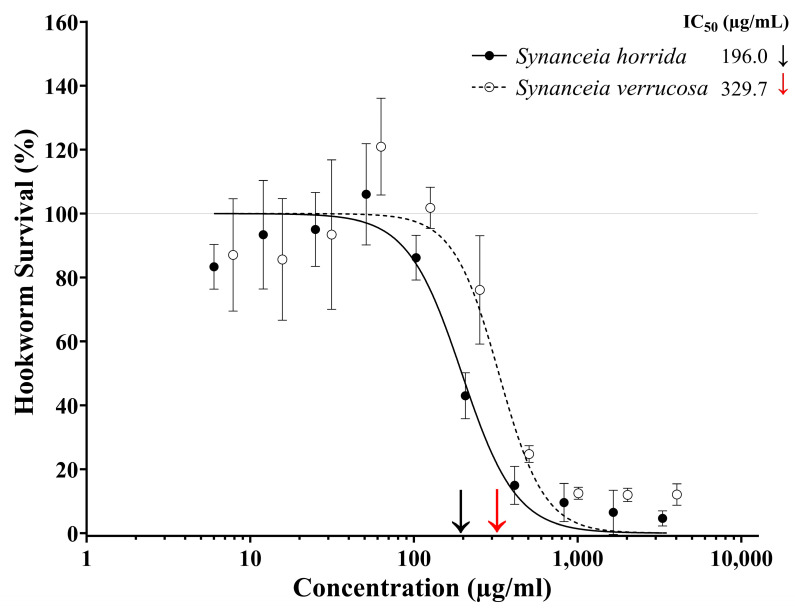
Dose-dependent effect of crude stonefish (*Synanceia* spp.) ichthyocrinotoxins on hookworm larvae survival. The motility of *N. brasiliensis* L3 (infective larval stage 3 Rodent Hookworm) relative to the negative (i.e., no toxin) control group has been used as an indicator for the percentage of hookworm larvae that survived following one hour of exposure to varying concentrations of *Synanceia horrida* (Estuarine Stonefish, ―●―) or *Synanceia verrucosa* (Reef Stonefish, ---○---) ichthyocrinotoxins. The IC_50_ values for each species toxin were calculated from the fitted curve and marked on the x-axis with **↓** for *S. horrida*, and **↓** for *S. verrucosa*. Error bars represent 95% confidence intervals.

A four-parameter logistic regression model (log inhibitor vs. normalised response, variable slope) was used to investigate the dose-dependent effect of crude stonefish ichthyocrinotoxins on the survival of *N. brasiliensis* L3 after one hour of exposure to treatment (Figure 1). A strong inhibitory dose–response relationship was observed for both *S. horrida* (Figure 1 R^2^ = 0.952, AICc = 199.5, HillSlope = −2.60) and *S. verrucosa* (Figure 1, R^2^ = 0.893, AICc = 214.1, HillSlope = −3.11) ichthyocrinotoxin treatments. A two-tailed *t*-test revealed a significant difference in the IC_50_ values between *S. horrida* and *S. verrucosa* ichthyocrinotoxins (*t*_0.05(2),36_ = 4.32, *p* = 0.0001), indicating that the anthelmintic activity of these toxins varies between the species. Specifically, ichthyocrinotoxin from *S. horrida* was significantly more toxic to *N. brasiliensis* L3 (IC_50_ = 196.0 µg/mL, 95% CI: 175.1–221.4 µg/mL) compared to ichthyocrinotoxin from *S. verrucosa* (IC_50_ = 329.7 µg/mL, 95% CI: 279.9–391.5 µg/mL).

### 2.2. Identifying Components in Stonefish Ichthyocrinotoxins with Anthelmintic Activity

**Figure 2:**
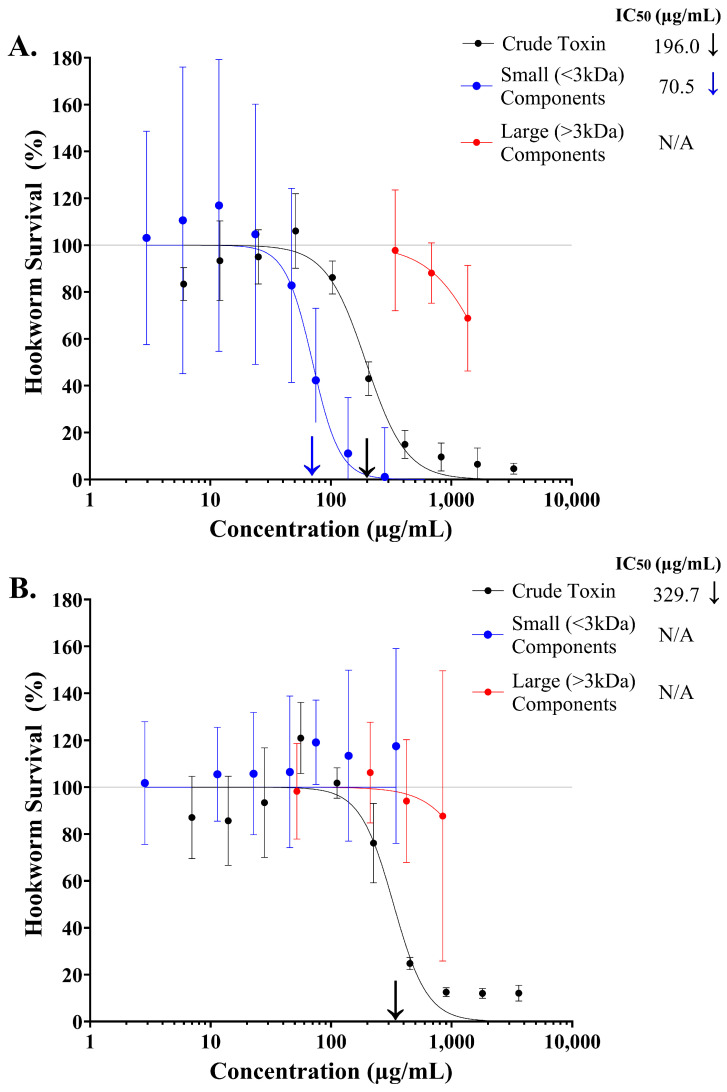
The anthelmintic activity of the isolated components from stonefish (*Synanceia* spp.) ichthyocrinotoxins. Dose–response analysis was used to determine the anthelmintic activity of the small (<3 kDa, ―●―) and large (>3 kDa, ―●―) components isolated from *Synanceia horrida* (Estuarine Stonefish, Panel (**A**)) and *S. verrucosa* (Reef Stonefish, Panel (**B**)) ichthyocrinotoxins against crude toxin (i.e., unprocessed, ―●―). Hookworm motility relative to the negative (i.e., no toxin) control group has been used as an indicator for the percentage of *N. brasiliensis* L3 that survived following one hour of exposure to each treatment. Error bars represent 95% confidence intervals. Arrows mark the IC_50_ values for crude (**↓**) and isolated small components (**↓**) from stonefish ichthyocrinotoxins on *N. brasiliensis* L3.

A four-parameter logistic regression model (log inhibitor vs. normalised response, variable slope) was used to investigate the dose-dependent effect of the large (>3 kDa) and small (<3 kDa) components isolated from stonefish ichthyocrinotoxins on the survival of *N. brasiliensis* L3 after one hour of exposure to treatment (Figure 2). A strong inhibitory dose–response relationship was observed for the small (>3 kDa) components isolated from *S. horrida* ichthyocrinotoxin (Figure 2A, R^2^ = 0.78, AICc = 238.6, HillSlope = −3.83). Given that these small components constitute approximately 50% of the crude *S. horrida* ichthyocrinotoxin mixture, their potency to *N. brasiliensis* L3 (IC_50_ = 70.5 µg/mL, 95% CI: 55.9–92.6 µg/mL) was comparable to that of the crude toxin (IC_50_ = 196.0 µg/mL, 95% CI: 173.9–222.1 µg/mL). In contrast, much higher concentrations (ten times greater) of the large components isolated from *S. horrida* ichthyocrinotoxin were required to induce a modest 20% inhibitory effect on *N. brasiliensis* L3 survival (IC_20_ = 990.0 µg/mL, 95% CI: 806.2–1270.0 µg/mL, R^2^ = 0.74, AICc = 45.7, HillSlope = −1.85) compared to the crude toxin. While crude *S. verrucosa* ichthyocrinotoxin elicited a strong dose-dependent inhibitory effect on hookworm survival (IC_50_ = 329.7 µg/mL, 95% CI: 279.9–391.5 µg/mL), both the isolated large and small components had minimal effect across all tested treatment dosages (Figure 2B). Only high concentrations of the large components (>500 µg/mL) resulted in a slight decrease (~10%) in hookworm survival.

### 2.3. Assessing the Cytotoxicity of Crude Stonefish Ichthyocrinotoxins to Mammalian Cell Lines In Vitro

**Figure 3:**
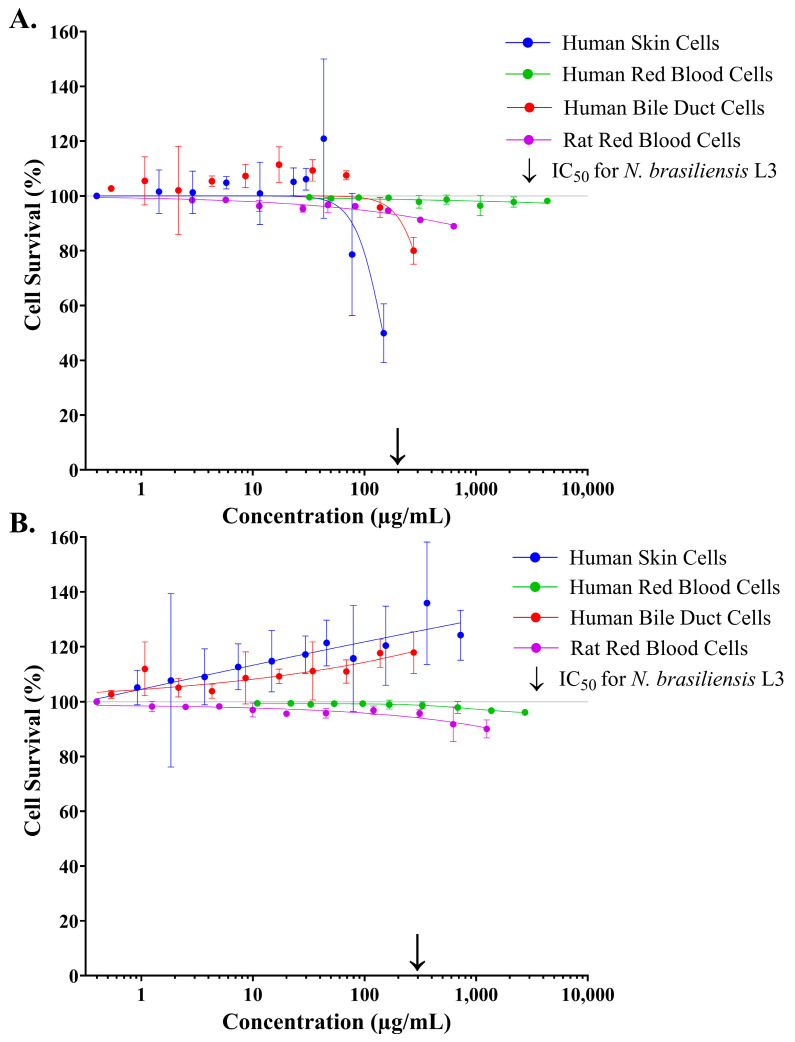
The effect of crude stonefish (*Synanceia* spp.) ichthyocrinotoxins on the survival of several mammalian cell lines maintained *in vitro*. Percent survival (%) of Human Normal Dermal Fibroblast Cells (―●―), Human Bile Duct Epithelial Cells (―●―), Human Red Blood Cell Erythrocytes (―●―), and Rodent Red Blood Cell Erythrocytes (―●―) was assessed one hour after the administration of crude *Synanceia horrida* (Estuarine Stonefish, Panel (**A**)) or *Synanceia verrucosa* (Reef Stonefish, Panel (**B**)) ichthyocrinotoxin at varying concentrations. Cell adhesion relative to the negative (i.e., no toxin) control group has been used as an indicator for the percentage of cells that survived following one hour of exposure to treatment. Arrows (↓) mark the IC_50_ values for crude stonefish ichthyocrinotoxins on *N. brasiliensis* L3. Error bars represent 95% confidence intervals.

A four-parameter logistic regression model (log inhibitor vs. response, variable slope) was used to investigate the dose-dependent effect of crude stonefish ichthyocrinotoxins on the survival of several mammalian cell lines in vitro, following one hour of exposure to treatment (Figure 3). Both *S. horrida* (Figure 3A) and *S. verrucosa* (Figure 3B) ichthyocrinotoxins had minimal effect on the survival of human red blood cells across all tested concentrations (*S. horrida*: R_2_ = 0.32, AICc = 2.35, HillSlope = −0.23, and *S. verrucosa*: R^2^ = 0.88, AICc = −42.25, HillSlope = −1.570). Similarly, both species toxins also had minimal effect on rodent red blood cells (*S. horrida*: R_2_ = 0.90, AICc = 6.12, HillSlope = −0.41, and *S. verrucosa*: R^2^ = 0.80, AICc = 29.37, HillSlope = −0.399); however, high treatment concentrations (>250 µg/mL) elicited a non-significant decrease in survival (up to 10%). Additionally, crude *S. verrucosa* ichthyocrinotoxin exhibited no inhibitory effect on the survival of human skin cells (R^2^ = 0.66, AICc = 141.6, HillSlope = 0.068) or human bile duct cells (R^2^ = 0.65, AICc = 94.03, HillSlope = 0.22) across all tested concentrations. Instead, the toxin appeared to stimulate the proliferation of both cell types, with a maximum effect of approximately 40% and 20% for skin and bile duct cells, respectively. In contrast, crude *S. horrida* ichthyocrinotoxin exhibited a strong dose-dependent inhibitory effect on the survival of human skin cells (R^2^ = 0.75, AICc = 135.9, HillSlope = −3.479), with an IC_50_ of 146.3 µg/mL (95% CI: 128.7–170.7 µg/mL). While crude *S. horrida* ichthyocrinotoxin also exerted an inhibitory effect on human bile duct cell survival (R^2^ = 0.43, AICc = 129.5, HillSlope = −3.361), its potency against these cells was notably lower, requiring a concentration of 274.6 µg/mL (95% CI: 242.5–324.9 µg/mL) to achieve only a 20% reduction in survival. Furthermore, at a concentration that resulted in 50% parasite mortality (IC_50_), crude *S. verrucosa* ichthyocrinotoxin had no negative effect on the survival of human bile duct and skin cells and had minimal effect on human and rat red blood cells. Similarly, crude *S. horrida* ichthyocrinotoxin exerted a minimal effect (>10% reduction in survival) on the survival of human or rat red blood cells, or human bile duct cells at the concentration that resulted in 50% hookworm death. In contrast, *S. horrida* ichthyocrinotoxin exerted a similar toxic effect on human skin cells, as it did on *N. brasiliensis* L3.

### 2.4. Assessing the Cytotoxicity of Isolated Small Components from Estuarine Stonefish Ichthyocrinotoxins to Mammalian Cell Lines In Vitro

**Figure 4:**
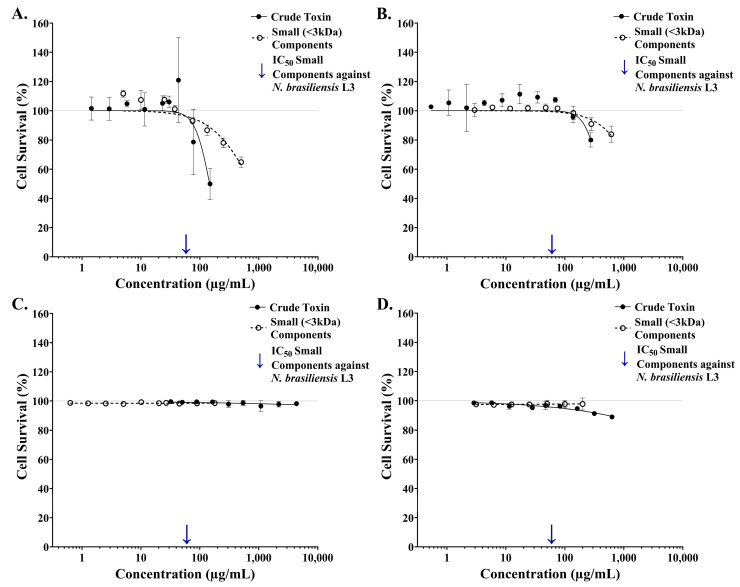
The toxicity of isolated small components from Estuarine Stonefish (*Synanceia horrida*) ichthyocrinotoxin to mammalian cell lines *in vitro*. Cell adhesion relative to the negative (i.e., no toxin) control group as an indicator of the percentage of Human Normal Dermal Fibroblast Cells (Panel (**A**)), Human Bile Duct Epithelial Cells (Panel (**B**)), Human Red Blood Cell Erythrocytes (Panel (**C**)), and Rodent Red Blood Cell Erythrocytes (Panel (**D**)) that survived following one hour of exposure to varying concentrations of crude toxin (―●―) and isolated small (<3 kDa) components (- - -○- - -) from *Synanceia horrida* (Estuarine Stonefish) ichthyocrinotoxin. Arrows (↓) mark the IC_50_ value of the small components isolated from *S. horrida* ichthyocrinotoxin against *N. brasiliensis* L3. Error bars represent 95% confidence intervals.

A four-parameter logistic regression model (log inhibitor vs. normalised response, variable slope) was used to determine the dose-dependent effect of the small components isolated from *S. horrida* ichthyocrinotoxin on the survival of several mammalian cell lines in vitro following one hour of exposure to treatment (Figure 4). Like the crude toxin, the isolated small components (<3 kDa) from *S. horrida* ichthyocrinotoxin had minimal effect on the survival of human or rat red blood cell erythrocytes, with ~99% cell survival observed across all tested concentrations (Figure 4C,D). Conversely, a strong inhibitory dose–response relationship was observed for the survival of human skin cells (Figure 4A, R_2_ = 0.82, AICc = 96.4, HillSlope = −1.25) and human bile duct cells (Figure 4B, R_2_ = 0.86, AICc = 51.25, HillSlope = −1.44). However, the small components demonstrated a weaker inhibitory effect on the survival of both cell lines compared to the crude toxin. While accurate IC_50_ values for the effect of the small components on mammalian cell lines could not be calculated from the available data, the IC_20_ was determined to be 250.4 µg/mL (95% CI: 204.6–314.0 µg/mL) for human skin cells and 706.9 µg/mL (95% CI: 628.9–807.3 µg/mL) for bile duct cells. Furthermore, at a concentration that resulted in 50% mortality of *N. brasiliensis* L3, the isolated small components from *S. horrida* ichthyocrinotoxin had minimal effect on the survival (>10% reduction in survival) of all tested cell lines.

### 2.5. Comparing the Toxicity of Crude and Isolated Small-Component Samples from Estuarine Stonefish Ichthyocrinotoxins Between Parasite and Cellular Targets

**Figure 5:**
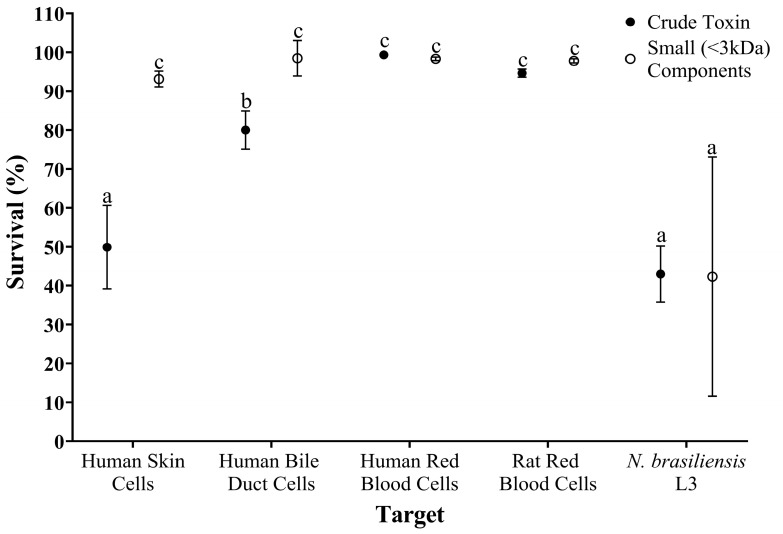
Comparative toxicity assessment of crude and isolated small components samples from Estuarine Stonefish ichthyocrinotoxin between parasite and cellular targets. Parasite motility (*N. brasiliensis* L3) and the adhesion of various mammalian cell lines (human skin and bile duct cells, human and rat red blood cells) relative to a negative (i.e., no toxin) control group as an indicator for the survival of parasites/cells following one hour of exposure to crude toxin (●) and isolated small (<3 kDa) components (○) from *Synanceia horrida* (Estuarine Stonefish) ichthyocrinotoxin. For isolated small components, the highest achievable treatment concentration shared by all targets (100 µg/mL) was selected for comparison. For crude toxins, a treatment concentration of 200 µg/mL was chosen for comparison to account for the estimated 50% composition of small components. Target survival is shown relative to the negative control group (no toxin exposure). Letters (a–c) indicate statistically significant post hoc LSD groupings. Error bars represent 95% confidence intervals.

The toxicity of crude and isolated small components from *S. horrida* ichthyocrinotoxin were compared between parasite (*N. brasiliensis* L3) and various cellular (skin, bile duct, and red blood cell) targets using two-way ANOVA (Figure 5). There was a significant interaction between toxin type (isolated small components vs. crude) and target (*N. brasiliensis* L3, human skin cells, etc.), on survival (*F*_4,23_ = 10.89, *p* < 0.001). There were also significant effects of target (*F*_4,23_ = 75.78, *p* < 0.001) and sample type (*F*_1,23_ = 26.76, *p* < 0.001) on survival.

Post hoc LSD revealed that the small components isolated from *S. horrida* ichthyocrinotoxin had little effect on the survival of mammalian cells, with all cell lines displaying statistically similar survival rates exceeding 95% (±2%). In contrast, the small components exhibited a significantly greater toxicity against *N. brasiliensis* L3 (~50% ± 10% decrease in survival) compared to mammalian cells. Furthermore, crude toxin also exerted minimal effects on the survival of human and rat red blood cells (~99% and 95% ± 2% survival, respectively) and had only a mild effect (~20% ± 5% decrease in survival) on human bile duct cells. In contrast, the effect of crude toxin on the survival of human skin cells was comparable to that observed for *N. brasiliensis* L3, with a ~50% ± 10% decrease in survival observed across both targets.

## 3. Discussion

The present study investigated the anthelmintic activity of stonefish ichthyocrinotoxins and their potential applications as novel therapeutics. Stonefish ichthyocrinotoxins were found to elicit a dose-dependent toxic effect on *N. brasiliensis* L3. These findings further support the hypothesis that stonefish ichthyocrinotoxins may function, at least in part, in parasite defence. Additionally, previous research has shown that stonefish ichthyocrinotoxins also exhibited toxicity towards ciliated protozoans [20]. Together, these findings suggest that stonefish ichthyocrinotoxins may provide protection against a broad spectrum of parasite taxa, ranging from single-celled protozoa to more complex multicellular organisms like nematodes.

Interestingly, the potency of stonefish ichthyocrinotoxins to *N. brasiliensis* L3 differed between the two species. Specifically, crude ichthyocrinotoxin from *S. horrida* was found to be significantly more toxic to the parasite (IC_50_ = 196.0 µg/mL) than ichthyocrinotoxin from *S. verrucosa* (IC_50_ = 329.0 µg/mL). The components responsible for the anthelmintic activity were also found to differ between the two species. The anthelmintic properties of *S. verrucosa* ichthyocrinotoxin were lost upon separation of its larger and smaller components. This suggests that the anthelmintic activity of *S. verrucosa* ichthyocrinotoxin either arises from synergistic interactions (i.e., the combined action of multiple components of different sizes), or that the activity was lost during sample processing. In contrast, most of the anthelmintic activity of *S. horrida* ichthyocrinotoxin was associated with its smaller components. However, it remains unclear whether this activity is due to a single active compound or synergistic interactions between compounds.

Previous research has shown that *S. horrida* ichthyocrinotoxin has negligible protease, haemolytic, and haemagglutinating activities [20]. Conversely, *S. horrida* ichthyocrinotoxin has also been shown to inhibit contractions in guinea pig ileum and vas deferens [20,30]. Our study provides further support for the varied cytotoxic properties of stonefish ichthyocrinotoxins. Crude ichthyocrinotoxins from both *S. horrida* and *S. verrucosa* had little to no effect on human and rat red blood cells, even at high concentrations (>250 µg/mL). Additionally, ichthyocrinotoxin from *S. verrucosa* appeared to promote the proliferation of human skin and bile duct cells. In contrast, crude ichthyocrinotoxin from *S. horrida* was highly toxic to human skin cells (IC_50_ = 146.3 µg/mL) and exhibited mild toxicity toward human bile duct cells (IC_20_ = 274.6 µg/mL).

The observed interspecific differences in the anthelmintic activity and cytotoxicity of stonefish ichthyocrinotoxins are consistent with previously reported compositional variations [31]. It was suggested that interspecific compositional variation in stonefish ichthyocrinotoxins may be driven by differences in the ecology of these species [31]. While the biological significance of the observed cytotoxicity to some mammalian cell lines remains unclear, interspecific variation in the toxins’ anthelmintic activities may be driven by differing parasite dynamics in the respective habitats of these species. For instance, the greater anthelmintic potency of *S. horrida* ichthyocrinotoxin may reflect a higher abundance and/or diversity of parasites in estuarine habitats compared to coral reefs. Alternatively, the parasite taxa that these species encounter may be different between reef and estuarine environments, necessitating different compounds for an effective parasite defence. It is also plausible that the increased connectivity between terrestrial and estuarine environments could enhance the exposure of primarily estuary-dwelling stonefish species, such as *S. horrida*, to mammalian-hosted parasites. This potential exposure may also contribute to the observed interspecific differences in the anthelmintic activity of stonefish ichthyocrinotoxins to *N. brasiliensis* L3. However, it is important to note that *N. brasiliensis* is not a natural parasite of stonefish. Therefore, the observed differences in the anthelmintic properties between stonefish species might be an artefact rather than a reflection of true ecological scenarios.

Nevertheless, the observed anthelmintic activity of stonefish ichthyocrinotoxins towards a mammalian-hosted nematode suggests potential applications beyond their immediate ecological context, such as a novel anthelmintic therapeutic. From this perspective, ichthyocrinotoxin from *S. horrida* emerges as a more attractive candidate for therapeutic development due to the small size of the active component(s). Small molecules are often preferred for drug discovery due to their ease of characterisation, lower manufacturing costs, enhanced stability, and more predictable pharmacokinetic and pharmacodynamic properties [32,33]. In clinical settings, this may translate to greater affordability for patients and healthcare systems, oral treatment options, and simpler dosing regimens [32,33]. In contrast, due to the potential synergistic nature of its anthelmintic activity, *S. verrucosa* ichthyocrinotoxin may pose greater challenges for downstream development and clinical implementation.

The therapeutic potential of the isolated small components from *S. horrida* ichthyocrinotoxin is further strengthened by their high potency against *N. brasiliensis* L3 and reduced toxicity to mammalian cells. At a concentration of 100 µg/mL, these components caused less than 10% cell death in all tested mammalian cell lines but inhibited *N. brasiliensis* L3 survival by over 50%. Additionally, while the small components did exhibit some dose-dependent toxicity to human skin and bile duct cells, they were significantly less cytotoxic than the crude toxin. Nearly twice the concentration of the small component sample was required to reduce the survival of human skin cells by only 20% (IC_20_ = 250.4 µg/mL) compared to the concentration of crude toxin that halved cell survival (IC_50_ = 146.3 µg/mL). Similar results were observed for bile duct cells, where more than twice the concentration of small components (IC_20_ = 706.9 µg/mL) was needed to achieve the same effect as the crude toxin (IC_20_ = 274.6 µg/mL). Furthermore, at biologically comparable concentrations, the small components demonstrated a weaker dose-dependent effect on human skin and bile duct cell survival compared to the crude toxin. This suggests that the cytotoxicity of *S. horrida* ichthyocrinotoxin is either driven by synergistic interactions between its small and large components, or that the toxins activity was lost through fractionation. Furthermore, the components responsible for the observed cytotoxicity may differ from those active against *N. brasiliensis* L3, potentially making them ideal candidates for further in vivo evaluation.

Despite showing promise for therapeutic development, the isolated small components from *S. horrida* ichthyocrinotoxin exhibit considerably lower in vitro potency to parasites (IC_50_ = 65.80 µg/mL) compared to established anthelmintics like ivermectin [34,35]. However, the isolated small components from *S. horrida* ichthyocrinotoxin are likely a multifarious mixture; thus, further isolation, as well as the structural and functional characterisation of the active component(s), is necessary to determine their true potency and clinical relevance. Additionally, future studies should determine the efficacy of these toxins against a wider range of parasite species, particularly medically significant hookworm species such as *Necator americanus* (human hookworm) and *Ancylostoma ceylanicum* (canine and human hookworm), to evaluate their broad-spectrum potential.

## 4. Conclusions

In conclusion, this study demonstrates the anthelmintic properties of stonefish ichthyocrinotoxins and highlights their potential as novel therapeutics. While both *S. horrida* and *S. verrucosa* ichthyocrinotoxins showed activity against skin-burrowing hookworm larvae, the isolated small component mixture from *S. horrida* ichthyocrinotoxin emerged as the most promising drug lead due to its increased potency to the parasite, favourable size for drug development, and reduced cytotoxicity compared to the crude extract. However, further isolation and identification of the active component(s) within *S. horrida* ichthyocrinotoxin are needed to fully determine its clinical relevance.

## 5. Materials and Methods

### 5.1. Ichthyocrinotoxin Collection and Parasite Cultivation

#### 5.1.1. Ichthyocrinotoxin Collection

Ichthyocrinotoxins from *S. verrucosa* and *S. horrida* were collected following the protocol outlined by [31]. A single specimen (*S. horrida* or *S. verrucosa*, 100 to 400 mm body length) was transferred to a damp surface and secured by gently holding either side of the pectoral fins. A saltwater-dampened cloth was placed over the animal’s eyes to minimise stress throughout the process. Ichthyocrinotoxin was then extracted from the skin tubercles using targeted mechanical massage under a controlled vacuum. This vacuum was generated by a purpose-built extraction apparatus consisting of a 100 mm segment of clear vinyl tubing affixed to a 50 mL syringe. To account for potential individual variation in toxin composition, ichthyocrinotoxin was extracted and pooled from three to five individuals of each species. Pooled ichthyocrinotoxin samples were stored in 1.5 mL Eppendorf tubes at −80 °C until required. The concentration of crude ichthyocrinotoxin was determined for both species using a standard Pierce™ Bicinchoninic Acid (BCA) Protein Assay kit, following the manufacturer’s instructions.

#### 5.1.2. Parasite Cultivation

Infective stage larval instar three (L3) *Nippostrongylus brasiliensis* (Rodent Hookworm) were cultivated following a protocol adapted from Camberis, et al. [36]. Briefly, *N. brasiliensis* L3 were suspended in 1× Phosphate-Buffered Saline (PBS) at a density of 2500 to 3000 L3/250 µL. Two female Sprague–Dawley rats were then subcutaneously injected between the shoulder blades with 250 µL of the larval suspension in accordance with James Cook University Animal Ethics approval (A2626). The rats were then monitored daily for any physical or behavioural abnormalities, and on the fifth day post-infection, they were transferred to new cages with clean bedding. Infected faecal pellets were collected on the sixth and seventh days post-infection. The faecal pellets were saturated in reverse-osmosis (R/O) water and blended into a slurry. The slurry was then combined with granulated and fine particulate activated charcoal at a ratio of roughly 1 part faecal slurry: 2 parts granulated charcoal: 1 part fine particulate charcoal, or until the mixture resembled natural soil. This mixture was evenly spread across sterile 90 mm plastic petri dishes lined with R/O water-dampened filter paper (Whatman^®^ Buckinghamshire, United Kingdom, 80 mm rounded, Grade 4, 20–25 µm pore size). The Petri dishes containing the faecal culture were then set to incubate undisturbed in the dark at 26 °C for five days to allow the larvae to hatch and migrate to the periphery of the plates. *Nippostrongylus brasiliensis* L3 were gently washed from the plate sides and lid with a Pasteur pipette and R/O water. The suspension was transferred to a 50 mL Falcon tube and brought to the nearest 10 mL gradation with R/O water. To quantify the number of *N. brasiliensis* L3 harvested, 100 µL of larval suspension was diluted 1:10 with R/O water. Then, 100 µL of diluted larval suspension was aliquoted on to a clean 90 mm plastic Petri dish, and larvae were counted under a top light microscope. The total number of larvae harvested was extrapolated by multiplying counts by the dilution factor (DF = 100).

### 5.2. Measuring Hookworm Larvae Motility with xWORM

#### 5.2.1. Measuring Hookworm Larvae Motility

The effect of stonefish ichthyocrinotoxins on the motility of *N. brasiliensis* L3 was assessed using a modified version of xWORM (xCELLigence Worm Real-Time Motility Assay) adapted from [37]. Assay parameters including media type, media concentration, and in-well parasite density were selected for *N. brasiliensis* L3 based on the findings from Lennox-Bulow, et al. [38]. In brief, two 96-well E-plates were pre-loaded with 100 µL of 0.25× PBS and 2% Antibiotic–Antimycotic (AA) solution in each well. The E-plates were then placed on an xCELLigence Real-Time-Cell-Analyser (RTCA) single-plate (SP) instrument to undergo an initial background measurement. The xCELLigence SP instrument was incubated in a temperature-controlled cabinet maintained at 37 °C and 5% CO_2_ for the duration of the experiment to simulate the internal conditions of a mammalian host, and thus stimulate L3 motility. For each E-plate, a total of 48 wells were loaded with 50 µL of live *N. brasiliensis* L3 suspension (300 L3/50 µL of 0.25× PBS larval suspension), and a further 24 wells received 50 µL of heat-killed (95 °C for 5 min) L3 suspension at the same density. To mitigate the effect of media evaporation during incubation, parasites were only added to the inner wells of the E-plates. Wells along the outer perimeter of the plates received 50 µL 0.25× PBS with 2% AA and were excluded from subsequent analysis. The motility of *N. brasiliensis* L3, which was used as a proxy for hookworm survival, was continuously monitored at 25 kHz with regular user-defined readings taken every 15 s over a 72 h period. The larvae were acclimated to the experimental conditions for 20 h prior to the administration of treatment.

#### 5.2.2. Data Transformation to Motility Index/Percent Parasite Survival

Raw Cell Index readings from RTCA Software Pro version 2.0 were exported for processing into a motility index, a measure of the standard deviation of the difference from the rolling Cell Index mean over time, as described by [35]. To account for background noise, motility index data were blank-adjusted by subtracting each data point of each treatment group from the mean of its corresponding positive control group (i.e., heat-killed L3 that had received the same dosage of *S. horrida* or *S. verrucosa* ichthyocrinotoxin). Variations in baseline motility between live *N. brasiliensis* L3 wells were compensated for by identifying the highest motility value across all wells at a single pre-treatment time point (19 h post assay commencement) and dividing this by the motility index recorded for each individual well at that time point. This yielded an adjustment factor for each well, which was then applied by multiplying this factor by all proceeding motility index values (post-treatment). Adjusted motility index values were then converted to percent motility, herein referred to as hookworm survival, by calculating the relative motility index of each treatment group compared to the negative control (wells containing live L3 that received 0.25× PBS only) and multiplying by 100.

### 5.3. Assessing the Anthelmintic Activity of Stonefish Ichthyocrinotoxins and Identifying Hookworm-Toxic Components

xWORM and data transformation procedures detailed in Section 5.2 were employed for all experiments outlined in this section. To evaluate the effect of ichthyocrinotoxin samples on the survival of *N. brasiliensis* L3, we exposed the parasites to toxins for one hour prior to all subsequent analyses. This exposure duration was chosen to mimic a plausible infection scenario, as the parasite is expected to penetrate a host’s skin within this timeframe.

#### 5.3.1. Determining the Effect of Crude Stonefish Ichthyocrinotoxins on Hookworm Larvae Survival

To determine the effect of stonefish ichthyocrinotoxins on the survival of *N. brasiliensis* L3, crude samples of ichthyocrinotoxin from *S. horrida* and *S. verrucosa* were serially diluted (1 part toxin: 2 parts 0.25× PBS) to eleven concentrations that were four times more concentrated than the final in-well treatment concentration. Treatment involved the addition of 50 µL of crude 4× concentrated *S. horrida* or *S. verrucosa* ichthyocrinotoxin samples to 150 µL of parasite/0.25× PBS mixture (total well volume 200 µL). The final in-well ichthyocrinotoxin treatment concentrations applied to *N. brasiliensis* L3 ranged from 6 to 7200 µg/mL for both species’ toxins. Each condition (i.e., toxin concentration and stonefish species combination) was replicated four times on live parasite wells, which were assigned as the treatment group, and two times on heat-killed parasite wells, which served as a positive control group (i.e., 0% survival). An additional four wells containing live parasites served as the negative control group (i.e., 100% survival) and received 50 µL of 0.25× PBS with 2% AA in lieu of ichthyocrinotoxin. A four-parameter logistic regression model (log-inhibitor vs. normalised response, variable slope) was used to analyse the relationship between ichthyocrinotoxin concentration and *N. brasiliensis* L3 survival. Hookworm survival (%) one hour after receiving treatment was used as the dependent variable, log_10_-transformed ichthyocrinotoxin concentration was assigned as the independent variable, and stonefish species (*S. horrida* and *S. verrucosa*) that the ichthyocrinotoxin was sourced from was the covariate. The concentration of ichthyocrinotoxin required to inhibit parasite survival by 50% (IC_50_) was calculated for each stonefish species using the fitted relationship. A two-tailed *t*-test was then used to compare IC_50_ values between *S. horrida* and *S. verrucosa* ichthyocrinotoxin treatments following a method outlined by Motulsky [39].

#### 5.3.2. Isolating the Large (>3 kDa) and Small (<3 kDa) Components from Crude Toxin

To begin to identify the components responsible for the anthelmintic activity, crude ichthyocrinotoxin samples from *S. horrida* and *S. verrucosa* were each fractionated into two component groups: large (>3 kDa) components and small (<3 kDa) components. In brief, crude ichthyocrinotoxin (400 µL) was diluted in 4 mL of Milli-Q water and placed in the filter compartment of a centrifugal filter device (Amicon^®^ Millipore Ultra 15, Merck, Darmstadt Germany) with a 3 kDa molecular weight cutoff. The device was then centrifuged at 4000× *g* at 4 °C for 10 min intervals until the sample volume in the filter compartment was reduced to 100 µL. Both the retained sample (large components) and the filtrate (small components) were collected. The filtrate was subsequently lyophilised to remove excess water. Both large and small component samples were then diluted/rehydrated to a final volume of 500 µL each. Protein concentrations were quantified using a standard Pierce™ BCA Protein Assay kit, following the manufacturer’s instructions. The protein concentrations of the isolated small and large component groups were used to estimate their relative abundance within the original crude toxin mixture. The abundance of each component group within the crude toxin was determined to be approximately equal.

#### 5.3.3. Measuring the Dose-Dependent Effect of Isolated Toxin Components and Comparing Their Activity Against Crude Toxin

xWORM was used to determine whether the large and small components isolated from stonefish ichthyocrinotoxins exhibited a dose-dependent effect on the survival of *N. brasiliensis* L3. The small (<3 kDa) components isolated from stonefish ichthyocrinotoxins were serially diluted (1 part toxin: 2 parts 0.25× PBS) to yield nine treatment concentrations ranging from 3 to 600 µg/mL for *S. horrida*, and eight treatment concentrations ranging from 3 to 345 µg/mL for *S. verrucosa*. The large (>3 kDa) components isolated from stonefish ichthyocrinotoxins were also serially diluted (1 part toxin: 2 parts 0.25× PBS) to three treatment concentrations ranging from 341 to 1368 µg/mL for *S. horrida*, and four treatment concentrations ranging from 51 to 843 µg/mL for *S. verrucosa*. Treatment was administered to *N. brasiliensis* L3 as described in Section 5.3.1.

Separate four-parameter logistic regression models (log-inhibitor vs. normalised response, variable slope) for each species toxin (*S. horrida* and *S. verrucosa*) were performed to analyse the relationship between ichthyocrinotoxin concentration and *N. brasiliensis* L3 survival. Parasite survival (%) one hour after receiving treatment was used as the dependent variable, log_10_-transformed ichthyocrinotoxin concentration was assigned as the independent variable, and toxin type (isolated small components, isolated large components, crude toxin) was the covariate. The concentration of ichthyocrinotoxin required to inhibit parasite survival by 50% (IC_50_) was calculated for each toxin type and stonefish species using the fitted relationship.

### 5.4. Determining the Cytotoxicity of Stonefish Ichthyocrinotoxins to Mammalian Cell Lines In Vitro

#### 5.4.1. Culturing Mammalian Cell Lines In Vitro

Human normal dermal fibroblast (1BR.3.GN) cells were obtained from the European Collection of Authenticated Cell Cultures (ECACC, product code 90020509). The cholangiocyte cell line (H69), an SV40-transformed bile duct epithelial cell line derived from a non-cancerous human liver, was obtained in 2010 from Dr. Gregory J. Gores, Mayo Clinic, Rochester, Minnesota [40]. These cell lines will herein be referred to as human skin cells for 1BR.3.GN, and human bile duct cells for H69. Both cell lines were cultured according to manufacturer protocols. In brief, cells were seeded in 75 cm^2^ filter top flasks with 15 mL of an appropriate complete growth medium and incubated at 37 °C with 5% CO_2_. The complete growth medium for human skin cells consisted of DMEM/Nutrient Mixture F12 with 10% Foetal Bovine Serum (FBS) and 1% Antibiotic–Antimycotic (AA). The complete growth medium for human bile duct cells comprised high-glucose DMEM/Nutrient Mixture F12 with 10% FBS, 1% AA, Adenine (25 µg/mL), Insulin (5 µg/mL), Epinephrine (1 µg/mL), Holo-transferrin (8.3 µg/mL), Hydrocortisone (0.62 µg/mL), T3 enzyme mix (13.6 ng/mL), and Epidermal Growth Factor (10 ng/mL).

Cell lines were left to form a monolayer until reaching approximately 80% confluence before being passaged. Cells were then gently washed in 10 mL 1× PBS to remove debris, followed by detachment using 3 mL of 1× Tryple reagent (Gibco). Harvested cells were combined with 12 mL of their respective complete growth medium and centrifuged at 150× *g* for 5 min (Hettich ROTINA 420R Centrifuge). The resulting supernatant was discarded, and the remaining cell pellet was resuspended in fresh complete growth medium. The concentration of all cell lines was determined using a haemocytometer.

#### 5.4.2. xCELLigence Cell Viability Assay

The cytotoxicity of *S. horrida* and *S. verrucosa* ichthyocrinotoxin samples (crude and isolated small components) to human skin cells and human bile duct cells in vitro was assessed using a cell adhesion assay. Cell adhesion, which was used as a proxy for cell survival, was monitored in real-time using an xCELLigence RTCA (Agilent) system. E-Plates were loaded with 50 µL of the appropriate complete growth medium and then placed on an xCELLigence RTCA single-plate (SP) instrument to undergo an initial background measurement. Then, either 100 µL of human skin cell suspension or 100 µL of human bile duct cell suspension was aliquoted in each well of a 96-well E-plate at a density of 5000 cells/100 µL. Eight E-plates were set up with one cell type (human skin cells or human bile duct cells), stonefish species ichthyocrinotoxin (*S. horrida* or *S. verrucosa* ichthyocrinotoxin), and sample type (crude sample or isolated small components) combination allocated per plate. All E-plates were then incubated at 37 °C and 5% CO_2_ throughout the duration of the experiment to mimic the internal thermal conditions of a mammal. Cells were left to form a monolayer on the bottom of the wells for 19 h prior to the addition of treatment. Samples of crude *S. horrida* and *S. verrucosa* ichthyocrinotoxin and small (<3 kDa) components isolated from *S. horrida* ichthyocrinotoxin were prepared by serial dilution (1 part toxin: 2 parts complete growth medium). Treatment involved the addition of 50 µL of 4× concentrated samples of crude or isolated small components from *S. horrida* or *S. verrucosa* ichthyocrinotoxin to wells containing 150 µL of human skin or bile duct cells in their relevant complete growth medium. The final in-well treatment conditions were as follows: eleven concentrations of crude samples from *S. horrida* or *S. verrucosa* ichthyocrinotoxin ranging from 0.9 to 725 µg/mL were applied to human skin cells; ten concentrations of crude samples of *S. horrida* or *S. verrucosa* ichthyocrinotoxin ranging from 0.5 to 275 µg/mL were applied to human bile duct cells; eight concentrations of isolated small components samples from *S. horrida* ichthyocrinotoxin ranging from 5 to 600 µg/mL were applied to human skin cells; and nine concentrations of isolated small components samples from *S. horrida* ichthyocrinotoxin ranging from 3 to 700 µg/mL were applied to human bile duct cells. Each treatment condition was conducted in triplicate. To establish a reference for 100% cell survival, three wells on each E-plate were designated as a negative control group and received 50 µL of the relevant complete growth medium in lieu of ichthyocrinotoxin. Following treatment, cell progression was continuously monitored over 72 hours using xCELLigence RTCA (Agilent). The system performed 50 sweeps of the E-plate at intervals of 15 s for the first hour, 2 min for the second hour, 15 min for six hours, and 60 min thereafter, generating a continuous Cell Index trace (RTCA Software Pro version 2). Pre-treatment (19 h post assay commencement) Normalised Cell Index values were used to quantify the percent change in cell adhesion in response to treatment. Percent cell survival was calculated by dividing the Normalised Cell Index values of each treatment condition by the average normalised Cell Index value of the negative control group and multiplying by 100. Percent survival data were then exported to GraphPad Prism version 10 for dose–response analysis.

#### 5.4.3. Haemotoxicity Assay

The haemotoxicity of *S. horrida* and *S. verrucosa* ichthyocrinotoxin to human and rat red blood cell erythrocytes was assessed using an absorbance-based lysis assay outlined by Brinkman, et al. [41]. Rat blood was collected via cardiac puncture from two female Sprague–Dawley rats that had been recently euthanised (James Cook University Animal Ethics Approval A2626). Human blood was drawn from the vein (venipuncture) of a healthy donor with human research and ethics approvals provided by James Cook University (James Cook University Human Ethics Approval H8523). To isolate red blood cell erythrocytes, whole-blood samples were centrifuged at 3000× *g* for 10 min at 4 °C. Following centrifugation, the resulting supernatant was discarded, and the sedimented erythrocytes were resuspended in sterile 1× PBS. This process was repeated five times to thoroughly wash the erythrocytes and ensure the removal of any remanent plasma. A 1% erythrocyte suspension (approximately 42 × 10^6^ cells/450 µL), one each for human and rat erythrocytes, was prepared in 1× PBS. Human and rat erythrocyte suspensions (450 µL) were each aliquoted into 1.5 mL Eppendorf tubes.

Treatment involved the addition of 150 µL of crude *S. horrida* or *S. verrucosa* ichthyocrinotoxin, or isolated small (<3 kDa) components from *S. horrida* ichthyocrinotoxin at various concentrations to individual 1.5 mL Eppendorf tubes containing 450 µL of human or rat red blood cell erythrocytes (*n* = 51 tubes). Samples of crude *S. horrida* and *S. verrucosa* ichthyocrinotoxin and isolated small components from *S. horrida* ichthyocrinotoxin were first prepared by serial dilution (1 part toxin: 2 parts 1× PBS) to a series of samples that were 4 times more concentrated than the final in-well treatment conditions. The following final in-well treatment concentrations were tested for haemolytic activity to human and rat red blood cell erythrocytes: ten concentrations of crude *S. horrida* or *S. verrucosa* ichthyocrinotoxin ranging from 1 to 1243 µg/mL were administered to rat red blood cells; eight concentrations of crude *S. horrida* or *S. verrucosa* ichthyocrinotoxin ranging from 10 to 4400 µg/mL were administered to human red blood cells; ten concentrations of isolated small component samples from *S. horrida* ichthyocrinotoxin ranging from 0.6 to 180 µg/mL were administered to human blood cells; and seven concentrations of isolated small components samples from *S. horrida* ichthyocrinotoxin ranging from 7.5 to 5000 µg/mL were administered to rat red blood cells. Additionally, one tube per cell type (i.e., human/rat cells) and treatment group (i.e., crude *S. horrida* ichthyocrinotoxin, isolated small components from *S. horrida* ichthyocrinotoxin, etc.) served as a positive control group (i.e., 100% cell lysis), which received 150 µL of lysis solution (1% Triton X-100 in 1× PBS) in lieu of toxin (*n* = 8 tubes). Another tube per cell type and treatment group was designated as a negative control group (i.e., 100% cell survival), which received 150 µL of 1× PBS (*n* = 8 tubes). All samples were then incubated for one hour at 37 °C with gentle agitation.

After the incubation period, samples were chilled on ice for 5 min, then centrifuged at 3000× *g* for 10 min at room temperature. Then, 200 µL of supernatant from each tube was transferred in triplicate to flat-bottomed 96-well plates. To account for background interference in absorbance readings, 200 µL of 1× PBS (media only wells) was aliquoted into three empty wells. Liberated haemoglobin was quantified by measuring its absorbance at 540 nm using a POLARstar Omega microplate reader. Raw absorbance data were blank adjusted by subtracting the average absorbance of the media-only wells. Percent lysis was determined by dividing each blank-adjusted absorbance value of the treatment group by the average of the positive control group and multiplying by 100. Percent survival was calculated by subtracting percent lysis from 100.

#### 5.4.4. Dose–Response Analysis—Mammalian Cell Lines

Separate four-parameter logistic regression models (log-inhibitor vs. response, variable slope) for each species toxin (*S. horrida* and *S. verrucosa*) were performed to analyse the relationship between crude ichthyocrinotoxin concentration and cell survival one hour after treatment administration. The same regression model was used to analyse the relationship between the concentration of the small (<3 kDa) components from *S. horrida* ichthyocrinotoxin administered and cell survival one hour post-treatment. Cell survival (%) one hour post-treatment was used as the dependent variable, log_10_-transformed ichthyocrinotoxin concentration was used as the independent variable, and cell type (i.e., human skin cells, human bile duct cells, human red blood cells, and rat red blood cells) was assigned as the co-variate. Where possible, the half maximal inhibitory concentration (IC_50_) of ichthyocrinotoxin for cell survival was calculated using the fitted relationship. In cases where only mild cytotoxicity was observed (i.e., where cell survival remained above 50%), the treatment concentration that resulted in a 20% inhibition (IC_20_) in cell survival was determined using a nonlinear regression model (log agonist vs. response—Find ECanything) with the following variables as constants: HillSlope = −1.0, F = 80, Bottom = 0, Top = 100.

### 5.5. Comparing the Activity of Crude- and Small-Component Samples from S. horrida Ichthyocrinotoxin Across Targets

The effects of crude toxin and isolated small (<3 kDa) components from *S. horrida* ichthyocrinotoxin on survival rates were compared between parasite and cellular targets using a two-way ANOVA (IBM SPSS version 27). Percent target survival after one hour of exposure to treatment served as the dependent variable, while target (human skin cells, human bile duct cells, human red blood cells, rat red blood cells, and *N. brasiliensis* L3) and toxin type (crude toxin, isolated small components) were assigned as the independent variables. For the isolated small components, the highest achievable treatment concentration shared by all targets (100 µg/mL) was selected for comparison. For the crude toxin, a treatment concentration of 200 µg/mL was chosen for comparison to account for the estimated 50% composition of small components. Prior to analysis, the assumptions of normality and homoscedasticity were confirmed using Kolmogrov–Smirnov and Levene’s Statistic, respectively. Following a significant ANOVA result, the post hoc least significant difference (LSD) was used to identify specific target (cells/parasites) and toxin type (crude/small component) groups that exhibited statistically significant differences in survival. Analyses were conducted in IBM SPSS version 10.

## Data Availability

The original contributions presented in this study are included in the article. Further inquiries can be directed to the corresponding author.
